# Investigating flock-associated mimicry: examining the evidence for, and drivers of, plumage mimicry in the greater and lesser necklaced laughingthrush

**DOI:** 10.1098/rsos.230976

**Published:** 2024-04-10

**Authors:** Kamal Raj Gosai, Liping Zhou, Yang Liu, Edward L. Braun, Rebecca T. Kimball, Scott K. Robinson, Aiwu Jiang, Eben Goodale

**Affiliations:** ^1^ Guangxi Key Laboratory of Ecology and Conservation, College of Forestry, Guangxi University, Nanning, Guangxi 530004, People's Republic of China; ^2^ Department of Environmental Science, Tri-Chandra Multiple Campus, Tribhuvan University, Kirtipur, Kathmandu 44600, Nepal; ^3^ Kunming Natural History Museum of Zoology, Kunming Institute of Zoology, Chinese Academy of Sciences, Kunming, Yunnan 650223, People's Republic of China; ^4^ State Key Laboratory of Biocontrol, School of Ecology, Sun Yat-sen University, Shenzhen, Guangdong 518107, People's Republic of China; ^5^ Department of Biology, University of Florida, Gainesville, FL 32611, USA; ^6^ Florida Museum of Natural History, University of Florida, Gainesville, FL 32611, USA; ^7^ Department of Health and Environmental Sciences, Xi’an Jiaotong-Liverpool University, Suzhou, Jiangsu 215123, People's Republic of China

**Keywords:** avian plumage mimicry, convergent evolution, interspecific social dominance mimicry (ISDM), mixed-species flocks, species associations, visual deception

## Abstract

Visual mimicry is less understood in birds than in other taxa. The interspecific social dominance mimicry (ISDM) hypothesis asserts that subordinate species resemble dominant ones to reduce aggression. Plumage mimicry has also been consistently noted in mixed-species flocks (MSFs), suggesting a connection to grouping behaviour, although it is unclear whether this is linked to ISDM. We studied greater necklaced laughingthrush (GNLT, *Pterorhinus pectoralis*) and lesser necklaced laughingthrush (LNLT, *Garrulax monileger*), which were recently placed in different genera. Measurements of 162 museum specimens showed LNLT converging in sympatry with GNLT in necklace colour, but diverging in necklace to body ratio, with proportionally smaller necklaces. The species were closely associated in six of seven MSF systems from Nepal to China. In a study of foraging behaviour in Nepal, aggression was rare between the species, LNLT followed GNLT and had lower foraging rates when further from GNLT. Our data suggest a link between this MSF-associated mimicry and ISDM, and that the subordinate LNLT may be the mimic and gain more from the resemblance. The species spend much time together in dense and poorly lit vegetation, where the LNLTs resemblance to GNLTs potentially allows them to forage closer to GNLTs than would be otherwise possible.

## 1. Introduction

Mimicry involves the convergence of phenotypic traits of different organisms over evolutionary time and is one of the most vivid examples of the power of natural selection [1]. Interspecific mimicry involves models, mimics that are benefitted by the mimicry, and sometimes third parties that are deceived [1,2]. In some types of mimicry, all these parties are clearly differentiated, such as in Batesian mimicry, a type of protective mimicry in which a chemically defended model is imitated by a non-defended mimic to avoid predators [3], or in aggressive mimicry, in which the mimic uses imitation of a non-threatening or attractive model to attract its own prey [4]. In other types of mimicry, there may be fewer interacting parties. For example, species converge on a phenotype in Mullerian mimicry, and there is therefore no distinction between model and mimic [5], while in interspecific social dominance mimicry (ISDM), a subordinate species uses mimicry to reduce aggression from a dominant one, with the model being the audience for the mimicry [6]. The diversity of taxa involved, roles they can play and sensory modalities belie simple generalizations about mimicry, and makes the search for the explanation of any one case a complex endeavour.

In analysing a putative case of mimicry, the association between species at different spatial scales can help to understand the species’ roles and the function of mimicry. At the largest scale, usually, the ranges of models and mimics are similar, or mimics have a range within that of the models, although there are some exceptions in which mimics can exist without models [3,7,8]. In any one location within the species’ ranges, the species may also group together (i.e. associate together in the same flock), especially when that reinforces the signal to third parties [5]. Indeed, mimicry could be considered a subset of aposematism, which is often associated with aggregation [9,10]. A driver of such aggregation could be that predators may become confused when many similar-looking individuals group together [11]. Once it has been confirmed how species overlap in their distributions and within habitats, then the exact nature of the behavioural interaction requires study. Sometimes such detailed work can show that multiple functions of mimicry are gained at one time. For example, a fish that imitates cleaner fish was found to get protective advantages from its appearance, because host fish do not tend to eat cleaners [12]. At the same time, it is also an aggressive mimic, attacking fish that are deceived by its innocuous appearance, and taking scales, mucous and even tissues from the other fish.

Plumage mimicry in birds is becoming recognized to be more extensive than once thought, as species that look similar to each other are not found to be close relatives through phylogenetic data [6]. At the same time, its function remains controversial. The typical kind of Batesian mimicry involving protective chemicals is not thought to be widespread in birds, except in the cases of a few species in Papua New Guinea that appear to derive their toxins from beetles [13,14]. Rather, ISDM has been suggested to be more widely applicable to many types of birds, allowing subordinate species to avoid attack from dominant competitors by appearing similar to the dominants [6]. Potential examples include friarbirds and orioles, which forage in the same fruiting trees [15], or woodpeckers, which may be aggressive against each other in protecting scarce nesting sites [16,17]. However, another interpretation of such cases of ‘competitive mimicry’, is that the dominant modelled species may be difficult to subdue by predators, and therefore mimicry is actually leading to the deception of the third-party predators [18–20]. Regardless of who is being ‘duped’, this kind of mimicry can be associated with grouping behaviour, as in the friarbird and oriole example, but need not be, depending on what resource is competed over—subordinate and dominant woodpeckers are not often grouped together when finding and using nest holes.

Avian plumage mimicry has also been repeatedly thought to be associated with mixed-species flocks (MSFs), and thus related to grouping. The first researcher to hypothesize that plumage mimicry was occurring in MSFs was Moynihan [21,22], who suggested that this mimicry could better allow interspecific sociality and communication. Responding to Moynihan, and viewing Moynihan’s suggestions as group selectionist and therefore unlikely, Barnard argued that convergence could be instead the result of an ‘oddity effect’ [23] in which individuals that look different from the other members of a group are selected by predators for attack [11,24]. An oddity effect might be more likely to evolve in some areas where there is one species that dominates flocks numerically like what occurs in Asia [25], providing a template on which other species could converge. From the empirical perspective, there have been many instances where birds that participate in MSFs have been hypothesized to look similar to each other; a previous study showed that for the majority of these cases, the birds do look more similar to each other than closely related species or others in the same habitat, at least to human eyes [26]. A recent article [27] has also hypothesized that plumage convergences in MSFs could even lead to speciation, if there are flock types in different areas that have distinct plumage colours (such as ‘yellow belly’ or ‘rufous-white’ flocks [28]) and then different populations of the same species come to resemble these different flock types. Nonetheless, it remains unclear whether flock-associated mimicry is a separate phenomenon from ISDM, or is closely connected to it.

Here, we study a pair of species in MSFs: greater necklaced laughingthrush (GNLT, *Pterorhinus pectoralis*) and the lesser necklaced laughingthrush (LNLT, *Garrulax monileger*; taxonomy follows The Clements World Bird Checklist at http://www.birds.cornell.edu/clementschecklist/). These species appear very similar, but the most recent phylogenies demonstrated that they belong to separate clades of babblers, which diverged 18–20 million years ago [29]. We investigated this phenomenon at three different scales: (i) we asked how co-occurrence in their distributional range affects similarities between the species; (ii) we investigated whether the species flock together at different locations; and (iii) we looked at behavioural interactions between the species, to see if they could help suggest what function mimicry plays. Our hypotheses were based on the ISDM phenomenon, with the underlying ideas that the smaller LNLT may be the mimic, using mimicry to escape aggression from GNLT, and that the two species forage and compete in close proximity to each other, albeit in thick and poorly lit vegetation, where it might be difficult to make fine visual distinctions (table 1). We made several predictions that would support our hypotheses. First, that LNLT would appear to be more similar to GNLTs in its necklace—the central element of resemblance between the species—in sympatry, that its necklace would be highly variable in allopatry, and that the two species would be more different in size in sympatry, consistent with the idea that they are direct competitors that have undergone character displacement [30]. Second, that the species would co-occur in MSFs. Third, that the species would mix together in MSFs, so their distances to conspecifics would be greater when associated together. Further, we predicted that they would forage more when associated than when apart, and especially when they were closer to the other species. Again, since LNLT would be expected to be the mimic, we predicted LNLT might benefit more than GNLT, and thus its foraging success would be a selective force driving mimicry in LNLT and reducing aggression from their foraging associate.

**Table 1.** Hypotheses that could explain mimicry or other resemblances between bird species in MSFs, and specific predictions for the study based on ISDM. ((*a*) Major categories of plumage mimicry in birds differ in what they predict about the direction of the mimicry (which species imitates, or comes to resemble) and the required interaction (in what way the species are expected to interact). (*b*) Given that the necklaced laughingthrushes (NLTs) are of different size and known to often forage together, we suspected that ISDM might be involved, and therefore made specific predictions for the study based on that hypothesis.)

hypothesis	directionality	interaction
**(*a*) major hypotheses that could explain mimicry in MSFs**		
increased communication	none; both species evolve to look more alike	species should group together in close proximity, close enough to see each other
oddity effect	none; both species evolve to look more alike	species should group together in close proximity so that they would be seen at once by a shared predator
chemical mimicry	chemically non-defended imitates chemically defended	mimicry does not depend on the two species grouping together
ISDM	subordinate imitates dominant to avoid dominant’s aggression towards it	species may or may not group together in close proximity, depending on the type of resource they compete over. If food resource, they should be foraging close enough to see each other
mimicry of dominant competitor for third parties	subordinate imitates dominant as protection against predators or other competing species	mimicry does not depend on the two species grouping together

**(*b*) hypotheses for NLTs based on ISDM, and predictions for this study**		
overall hypotheses	*H*_A_. the smaller and likely subordinate LNLT is the mimic, and GNLT is the model	
	*H*_B_. the species are usually feeding together and competing at close range, although the thick vegetation and low light may make fine visual distinctions difficult	
plumage study predictions	LNLT will come to look more like GNLT in sympatry (based on *H*_A_) LNLTs in allopatry will be very variable (based on *H*_A_) the species are competitors, so in sympatry, they should be more morphologically dissimilar to avoid competition (i.e. there will be evidence of character displacement; based on *H*_B_).	
co-occurrence study predictions	species will be found in flocks more together than would be expected by chance (based on *H*_B_)	
foraging study predictions	species will mix together in flocks, so that they will be closer to conspecifics in monospecific flocks than in MSFs (based on *H*_B_) LNLT will forage more when in MSFs with GNLTs (based on *H*_A_) inside MSFs, LNLT will forage more when closer to GNLTs, and therefore LNLTs that are better imitators could be selected for (based on *H*_A_)	

## 2. Material and methods

### 2.1. Evaluation of plumage and morphological similarity in relation to co-occurrence

#### 2.1.1. Acquisition of images and measurements

**Figure 1.**
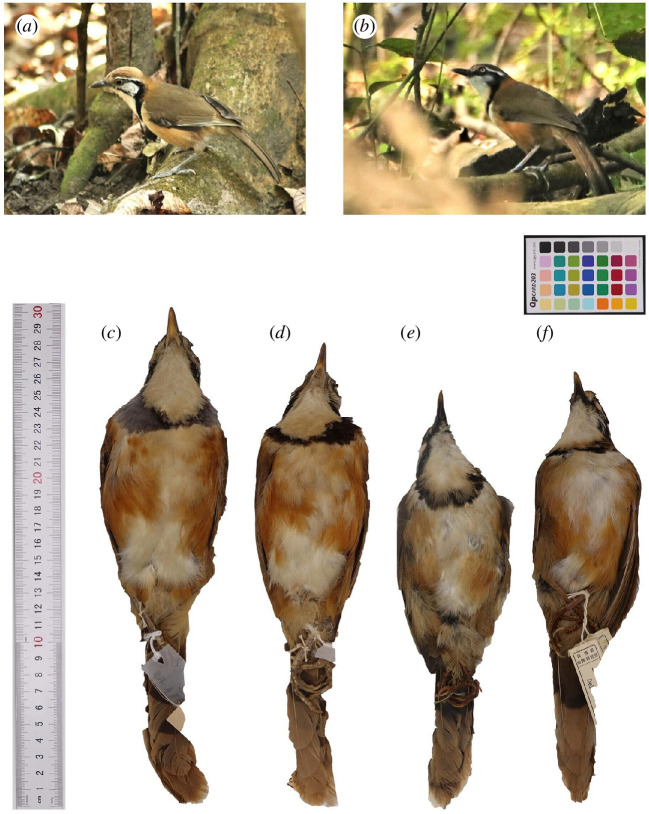
The two species as depicted by two photographs of Nepalese birds, and examples of Chinese museum specimens. Photographs in Chitwan National Park, Nepal, by K.R.G., depict (*a*) GNLT (*P. pectoralis*) and (*b*) LNLT (*G. monileger*). Photographs of the museum skins show variation in necklace colour within species, with (*c,d*) representing GNLT individuals with bright and dark necklace plumage, respectively, and (*e,f*) representing analogous LNLT individuals.

We visited three collections of bird specimens, the Kunming Natural History Museum (Kunming Institute of Zoology, Chinese Academy of Sciences, Kunming, China), the collection of the Department of Ecology, Sun Yat-sen University (Guangdong, China) and the Institute of Zoology (Chinese Academy of Sciences, Beijing, China), and took photographs and measurements of necklaced laughingthrushes (NLTs) skins. To take the photographs, the skins, facing upwards to display the necklace, were placed on a neutral background, with a colour card (Qp card 203) and ruler positioned beside the specimen inside a well-lit lightbox (figure 1). A camera (Canon 80D) was placed at a fixed distance (45 cm) above the skin with auto settings [31], and we took one picture per bird. At the same time, we took measurements of the length of the body (head to tail), beak, tail, tarsus and wing, with all measurements made by the same observer, K.R.G., using a digital vernier calliper. We also acquired photographs of skins (also with the same colour card and ruler beside the skin) and measurements from the Smithsonian National Museum of Natural History (Washington, DC, USA), Drexel University (Philadelphia, PA, USA) and National Museum of Natural History (Paris, France) of specimens that were collected either in China or Vietnam. All specimens had some information on the location (latitude and longitude) of where they were collected.

#### 2.1.2. Image processing

We measured the mean of red, green, blue (RGB) brightness values (R + G + B/3) of each photograph in two places: (i) the necklace of the specimen (S); and (ii) the blackest (B) square in the colour card. Dark-coloured specimens have a low value and light-coloured specimens have a high value (value 0: black/darkest; 255: white/brightest) of this measurement [31]. To control for variation in the incidence of light (especially for specimens not photographed in our light box), we then calculated the ratio of specimen plumage to the black in the colour card (S/B) as our metric of brightness, using ImageJ software (version ImageJ1.52a: Java1.8.0_112[64-bit]). Lower values of this ratio had darker plumage. Necklace size and area of body (body area from the line between the feet to the bill), both measured in cm^2^, were also calculated with the help of ImageJ (https://imagej.net/), by K.R.G.

#### 2.1.3. Classification of specimens

To delineate the distribution of the two species, we plotted the coordinates of the location where the specimen was collected in Google Earth (v. 7.3), and overlayed that with the distribution maps of NLTs provided by the Birds of the World (BoW; accessed electronically at https://birdsoftheworld.org). Skins of GNLT, which generally has a more northernly distribution, were classified as allopatric if they were from Anhui, Jiangsu, Guizhou, Hunan or Zhejiang Provinces, China, or in northern Yunnan Province close to Guizhou. It should be noted that there are some records of LNLTs in Guizhou and Zhejiang, but they are rare, and this is the species’ most northernly extreme, so abundances are presumably low. Skins of GNLTs were classified as sympatric if they were from Fujian, Guangdong, Guangxi, Hainan, Jiangxi and other parts of Yunnan. Similarly, allopatric LNLTs were defined as those from southern Vietnam, whereas sympatric LNLTs were from Fujian, Guangdong, Guangxi, Hainan, Yunnan and northern Vietnam. Altogether we analysed 162 skins (allopatric GNLTs: 20 skins; sympatric GNLTs: 77 skins; allopatric LNLTs: 11 skins; and sympatric LNLTs: 54 skins; electronic supplementary material, table S1; [32]). These skins were collected over a long-time range. Most of the specimens from China (85 of 137, 62.0%) were collected between 1951 and 1970, whereas the majority of the specimens from Vietnam (22 of 26, 84.6%) were collected between 1920 and 1940 (electronic supplementary material, table S2; [32]).

#### 2.1.4. Statistical analysis

We used several analyses to investigate changes in the plumage and morphology of the museum specimens. First, we wanted to see whether the species more resembled each other, or diverged from each other, in sympatry, and the directionality of this pattern; that is, which species appeared to change more. We compared allopatric to sympatric individuals of the same species (allopatric GNLT to sympatric GNLT, allopatric LNLT to sympatric LNLT), as well as contrasting allopatric individuals of the different species (allopatric GNLT to allopatric LNLT) and sympatric individuals of the different species (sympatric GNLT to sympatric LNLT), all with Welch’s *t*-tests for any particular trait. We identified convergence as occurring when the species more resembled each other in sympatry than in allopatry in the trait; divergence was the opposite pattern. We also compared the variation in the trait between allopatric and sympatric populations for each species with *F*-tests.

We also conducted a complementary ‘clinal analysis’ in which we performed a regression between species traits and latitude. Owing to gene flow, traits that imitate a model could be found in the absence of a model, especially in an area adjacent to a region of sympatry [7]. If a species changes in a trait to imitate another species, we might expect a cline in values of the trait that changes over distance, potentially within the region of sympatry (if, e.g. the abundance of models changes across that region) and outside of it; for this study, the most important direction is north-to-south, given that the regions of allopatry and sympatry are arranged in this way. This kind of clinal analysis has also been used to look at instances of trait divergence such as character displacement [33]. Another advantage of such a latitudinal analysis is we can compare trait distributions to well-known biogeographical patterns such as Bergmann’s rule (organisms are larger in colder environments [34]) and Gloger’s rule (organisms have darker colouration in more humid, and potentially warmer, environments [35]).

### 2.2. Co-occurrence of the species in mixed-species flocks

#### 2.2.1. Data acquisition

We analysed seven datasets about the two species’ occurrence in MSFs: three collected by other research teams, and used with their permission, and four we ourselves collected in the course of other research work. The datasets of other researchers included data from Arunachal Pradesh, India (provided by Umesh Srinivasan, *n* = 101 MSFs, as published in Srinivasan *et al*. [36]), and from the Nanling Mountains of northern Guangdong and southern Hunan, China (provided by Qiang Zhang, *n* = 341, as published in Zhang *et al*. [37]). Furthermore, we used data from north-central Myanmar (provided by David I. King, as published by King & Rappole [38]); but in this case, the researchers did not differentiate between the two species in the field, although they did report that both were present and estimated the relative abundance of the two species. As to the data we collected ourselves, two surveys were in the extreme southwestern portion of Yunnan, China, but covering slightly different study sites (*n* = 48 MSFs, collected by Y.L. and S.K.R. between 2017 and 2018; and *n* = 543 MSFs, collected by L.Z. in Tongbiguang Nature Reserve in 2021 [28]). In addition, we used a dataset from Zhejiang, China (*n* = 148 MSFs, collected by L.Z. in 2020 [39]), and one from Nepal (*n* = 328 MSFs, collected by K.R.G between 2020 and 2021 [40]; see below).

#### 2.2.2. Statistical analysis

Our analyses of co-occurrence assume that within a region both species could be found at any one location at any time. We used the Phi coefficient to judge the association between two species, which is based on the conventional 2 × 2 presence/absence tables, and calculated as:


$$Phi\;coefficient=(ad-bc)/[(a+b)\;(a+c)\;(b+d)\;(c+d){]}^{1/2},$$

where *a* is the number of times both species A and species B were present in a single MSF, *b* is the number of times only species A was present, *c* is the number of times only species B was present, and *d* is the number of times neither species A nor species B were present [41]. The Phi coefficient ranges from −1 (complete avoidance) to +1 (perfect co-occurrence). We tested the significance of the 2 × 2 table summarizing the presence and absence of one species, depending on the occurrence of the other species, using Fisher’s exact tests.

### 2.3. Association of the species in flocks of Nepal and their foraging ecology

#### 2.3.1. Field observations

In preliminary fieldwork, we visited various locations inside Chitwan National Park, an Important Bird Area of Nepal in the lowlands of the country, from October 2019 to January 2020, to assess sites where NLTs could be found [40]. Finding that the two species were observed regularly in the western area of the park (in the area between the villages of Pandavnagar and Golaghat; electronic supplementary material, figure S1 [32]), we placed seven 2 km transects on dirt roads and footpaths in the area, ensuring that they were at least 500 m apart at all points. In February and March 2019, in March and April 2021 and in March 2022, we visited these transects, between the times of 7.00–12.00 and 14.00–18.00. Each transect was visited only once a week; transects were visited at least three times in 2019 and 2021, and at least once in 2022.

Whenever we spotted a single-species or MSF with either species of NLTs, or both, we studied the flock from a distance of 15–20 m for 5–15 min and noted all the birds detected. If a flock had only one NLT species, we recorded the average distance between individuals of the species, and if a flock included both NLTs, we recorded the average distance between individuals of conspecifics, and also the average distance between individuals of the two species. We further looked for evidence of aggressive behaviours between NLTs, specifically looking for (i) chases between the two species; (ii) displacements, when one bird replaced another bird at a perch or foraging position (with the subordinate flying away); and (iii) deferments, when one bird tried a displacement, but the bird already there was dominant and did not move [42]. As for leadership, we looked for instances in which one species moved ahead of the flock (by at least 10 m), and then a heterospecific individual flew in the same direction (allowing the time of the second flight to be up to 2 min later), noting which species went first [43].

We also made observations of the foraging ecology of the birds. While observing MSFs during the 2021 and 2022 field seasons, we chose an individual of either species to observe, waiting a minute before collecting data so not to bias the data for locations or movements that were especially obvious [42]. In focal sampling [44], we then observed a bird for 2 min to note the number of prey items consumed. Both NLTs eat a variety of large insects (primarily caterpillars), so observations of prey consumption were possible. Simultaneously, we recorded how far the focal bird was to conspecifics and heterospecifics, as above.

#### 2.3.2. Statistical analysis

For this analysis, we used generalized linear mixed models to incorporate the units of replication, because multiple individuals could be observed in one MSF, and each flock was observed on a transect. We compared birds of one species when by themselves in a flock, or when associated with the other species of NLT in a MSF, in the distance to a conspecific (using a normal distribution) and foraging rate (using a Poisson distribution). We also constructed regressions for each species asking how the foraging rate (again modelled with a Poisson distribution) was influenced by distance to heterospecifics and distance to conspecifics. The random factors in these models were flock, nested in transect and year, and the models were run using the ‘lme4’ package [45] in the R statistical environment. When models were singular, indicating little variation explained by the random factors, we simplified the random factors (e.g. just flock), or even ran general linear models. Values of *p* ≤ 0.05 were considered significant. For measurements, means are shown ±s.d.; coefficients of models are shown ±s.e.

## 3. Results

### 3.1. Evaluation of plumage and morphological similarity in relation to co-occurrence

**Figure 2.**
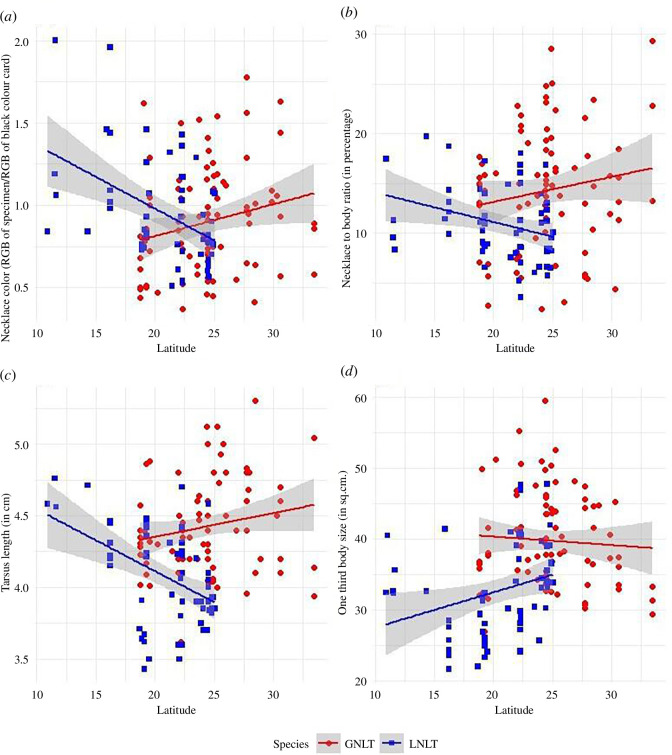
Significant results (*p* < 0.05) in clinal analysis of GNLT and LNLT traits across latitude: (*a*) necklace colour (a brightness measurement relative to the colour card), (*b*) necklace to body ratio, (*c*) tarsus length, and (*d*) body size.

Both *t*-tests (allopatry versus sympatry) and clinal analyses showed consistent results for some variables. First, sympatric LNLTs had darker necklace plumage than allopatric LNLTs (table 2); similarly, more northern LNLTs became darker in the clinal analysis (figure 2; table 3). Second, sympatric and more northern LNLTs had proportionally smaller necklaces relative to body size. Third, sympatric and more northern LNLTs had smaller tarsi. In other cases, the results of the *t*-tests and those of the clinal analyses were not consistently significant (southern GNLTs had darker necklaces and northern LNLTs had larger body size, but there were no differences in the respective *t*-tests; sympatric GNLTs had smaller tails, but there was no difference according to the clinal analysis).

**Table 2.** Nine kinds of measurements of museum specimens of the two species of laughingthrushes and the significance of comparisons, within and between species. (Abbreviations: Allop G, allopatric greater necklaced laughingthrush (GNLT); Symp G, sympatric GNLT; Allop L, allopatric lesser necklaced laughingthrush (LNLT); Symp L, sympatric LNLT. Sample sizes given in parentheses. *p*-values are for Welch’s *t*‐test, and significant values (*p* < 0.05) are bolded. Measurements in which there was evidence for convergence or divergence, based on differences between allopatric and sympatric specimens of the same species, are noted in the last (righthand) column.)

measurement	category	means values (±s.d.)	*t-*value	d.f.	*p*‐value	sympatry versus allopatry
necklace colour (RGB of specimen/RGB of black colour card)	Allop G (19)/Symp G (69)	0.98 ± 0.34/0.88 ± 0.32	1.17	27.27	0.25	convergence; LNLT giving significant change
	Allop L (10)/Symp L (45)	1.29 ± 0.42/0.87 ± 0.26	3.00	10.53	**0.020**	
	Allop G (19)/Allop L (10)	0.98 ± 0.34/1.29 ± 0.42	−2.09	15.51	0.061	
	Symp G (69)/Symp L (45)	0.88 ± 0.32/0.87 ± 0.26	0.022	107.48	0.98	
necklace size (cm^2^)	Allop G (19)/Symp G (58)	6.14 ± 2.53/5.71 ± 2.51	0.67	33.37	0.51	no significant change
	Allop L (11)/Symp L (40)	3.99 ± 1.21/3.30 ± 1.04	1.57	13.79	0.14	
	Allop G (19)/Allop L (11)	6.14 ± 2.53/3.99 ± 1.21	3.2	28.95	**0.003**	
	Symp G (58)/Symp L (40)	5.71 ± 2.51/3.33 ± 1.04	6.51	81.27	**<0.00001**	
necklace to body ratio (%)	Allop G (19)/Symp G (63)	15.73 ± 6.68/13.84 ± 5.98	1.13	27.71	0.27	divergence; LNLT giving a significant change
	Allop L (11)/Symp L (46)	13.27 ± 3.78/10.33 ± 3.52	2.32	14.31	**0.036**	
	Allop G (19)/Allop L (11)	15.73 ± 6.68/13.27 ± 3.78	1.3	27.95	0.2	
	Symp G (63)/Symp L (46)	13.84 ± 5.98/10.33 ± 3.52	3.86	103.22	**0.00020**	
tarsus length (cm)	Allop G (16)/Symp G (72)	4.51 ± 0.39/4.40 ± 0.30	1.02	19.33	0.32	divergence; LNLT giving a significant change
	Allop L (9)/Symp L (52)	4.47 ± 0.22/4.00 ± 0.29	5.73	14.43	**0.000046**	
	Allop G (16)/Allop L (9)	4.51 ± 0.39/4.47 ± 0.22	0.36	23	0.73	
	Symp L (46)/Symp G (72)	4.40 ± 0.30/4.00 ± 0.29	7.4	109.93	**<0.00001**	
wing length (cm)	Allop G (18)/Symp G (76)	12.82 ± 1.08/13.25 ± 0.75	−1.6	21.24	0.12	no significant difference
	Allop L (11/Symp L (51)	11.96 ± 0.96/11.85 ± 0.57	0.041	11.79	0.69	
	Allop G (18)/ Allop L (11)	12.82 ± 1.08/11.96 ± 0.96	2.33	23.79	**0.03**	
	Symp G (76)/ Symp L (51)	13.25 ± 0.75/11.85 ± 0.57	11.88	122.73	**<0.00001**	
beak length (cm)	Allop G (16)/Symp G (70)	2.76 ± 0.29/2.81 ± 0.24	−0.70	20.13	0.49	no significant difference
	Allop L (11)/Symp L (53)	2.43 ± 0.40/2.41 ± 0.20	0.10	11.20	0.92	
	Allop G (16)/Allop L (11)	2.76 ± 0.29/2.43 ± 0.40	2.48	17.44	**0.023**	
	Symp G (70)/Symp G (70)	2.81 ± 0.24/2.81 ± 0.24	11.88	122.73	**<0.00001**	
tail length (cm)	Allop G (16)/Symp G (72)	13.81 ± 0.59/13.37 ± 0.95	2.23	34.82	**0.02**	convergence, GNLT giving significant change
	Allop L (12)/Symp L (50)	12.72 ± 0.86/12.78 ± 0.85	−0.24	16.38	0.81	
	Allop G (16)/ Allop L (12)	13.81 ± 0.59/13.37 ± 0.95	3.79	18.36	**0.0013**	
	Symp G (72)/ Symp L (50)	12.72 ± 0.86/12.78 ± 0.85	3.57	112.9	**0.00053**	
body length (m)	Allop G (18)/Symp G (70)	30.16 ± 2.04/29.97 ± 2.10	0.36	27.69	0.72	no significant difference
	Allop L (12)/Symp L (50)	28.27 ± 1.82/27.54 ± 2.44	1.16	22.66	0.26	
	Allop G (18)/Allop L (12)	30.16 ± 2.04/28.27 ± 1.82	2.72	25.26	**0.011**	
	Symp G (70)/Symp L (50)	29.97 ± 2.10/27.54 ± 2.44	5.6	93.36	**<0.00001**	
body size (cm^2^)	Allop G (19)/Symp G (69)	38.39 ± 5.81/40.11 ± 6.95	−1.02	33.51	0.83	no significant difference
	Allop L (12)/Symp L (49)	31.17 ± 6.43/33.48 ± 7.14	−1.22	19.53	0.28	
	Allop G (19)/Allop L (12)	38.39 ± 5.81/31.17 ± 6.43	3.26	22.46	**0.0035**	
	Symp G (69)/Symp L (49)	40.11 ± 6.95/33.48 ± 7.14	4.95	99.24	**<0.00001**	

**Table 3.** Change in measurements of GNLT and LNLT across latitude. The coefficient for the regression line is given, as latitude gets more positive (northern). Significant (*p* < 0.05) regression results are bolded, and those that are similar to the allopatric versus sympatric test (table 2) are also italicized.)

		GNLT				LNLT			
	measurements	coefficient	s.e.	*F*-statistic	*p*‐value	coefficient	s.e.	*F*-statistic	*p*‐value
1	necklace colour (RGB)	0.020	0.0093	*F*_1,86_=4.31	**0.041**	−0.040	0.010	*F*_1,53_=14.16	***0.00042***
2	necklace size (sq. cm)	0.050	0.071	*F*_1,84_=0.46	0.49	−0.043	0.040	*F*_1,49_=1.17	0.29
3	necklace to body ratio (%)	0.25	0.18	*F*_1,80_=1.96	0.17	−0.29	0.12	*F*_1,55_=5.69	***0.021***
4	tarsus length (cm)	0.017	0.0093	*F*_1,86_=3.17	0.79	−0.043	0.010	*F*_1,59_=16.34	***0.00016***
5	wing length (cm)	−0.036	0.024	*F*_1,92_=2.17	0.14	0.022	0.021	*F*_1,60_=1.042	0.11
6	beak length (cm)	−0.013	0.0074	*F*_1,84_=2.92	0.091	0.0073	0.00064	*F*_1,62_=1.30	0.26
7	tail length (cm)	0.049	0.030	*F*_1,86_=3.43	0.067	0.040	0.030	*F*_1,60_=2.03	0.16
8	body length (cm)	0.080	0.060	*F*_1,86_=1.63	0.21	0.023	0.080	*F*_1,60_=0.10	0.77
9	one-third body size (sq cm)	−0.12	0.19	*F*_1,86_=0.39	0.54	0.10	0.21	*F*_1,58_=5.63	**0.021**

Concentrating on the consistent results, we can say that the changes in the LNLT populations led to convergence in necklace colour: sympatric LNLTs resembled closer GNLTs than allopatric populations (table 2). By contrast, changes in LNLTs led to divergence in proportional necklace size and tarsus size.

For GNLT, there was no difference in the variation in necklace characteristics between allopatric and sympatric populations (necklace colour: *F*_18,68_ = 1.15, *p* = 0.66; necklace size: *F*_19,57_ = 0.98, *p*=1.00; necklace to body ratio: *F*_18,62_ = 1.20, *p* = 0.58). However, for LNLT, there was more variation in necklace colour in allopatric populations, although the other variables did not show significant differences (necklace colour: *F*_9,44_ = 2.69, *p* = 0.03, necklace size: *F*_10,39_ = 1.54, *p* = 0.33; necklace to body ratio: *F*_10,45_ = 1.19, *p* = 0.65).

### 3.2. Co-occurrence of the species in mixed-species flocks

Neither species was very frequent in MSFs: in all seven datasets, the two species were always in a minority of the flocks of the regions, ranging between 5.4% and 35.2% of all MSFs (table 4). The species were usually tightly associated, with their co-occurrence patterns not independent of each other. In two datasets, the co-occurrence of the species was perfect: they were never found apart. In three other datasets, the species were non-randomly and positively associated. Only in the Arunachal Pradesh survey were the two species negatively associated (but there were only six MSFs in which one or the other was observed). The number of individuals of the two species per MSF was very variable: for example, there were 4.4 times as many LNLTs as GNLTs in Chitwan, Nepal (Welch’s *t*‐test, *t*_42.13_ = 10.67, *p* < 0.0001), but in the Nanling Mountains, China, there were 3.1 times more GNLTs than LNLTs (Welch’s *t*‐test, *t*_192.07_ = 12.81, *p* < 0.0001).

**Table 4.** MSF systems in locations across in Asia where both GNLT and LNLT are present. (Locations are ordered from west to east. Information is presented about the percentage of MSFs in which the species are present, their association in MSFs and their average number of individuals. For published references for the different locations (see §2.2.1).)

location of survey	MSFs with GNLT only	MSFs with LNLT only	MSFs with both present	MSFs with both absent	total	Phi coefficient	*p*‐value	% of MSFs with any NLT	avg individuals of GNLT	avg individuals of LNLT
Chitwan, Nepal	7	28	86	223	344	0.77	<0.0001	35.20	3.30 ± 1.60	14.50 ± 6.80
Arunanchal, India	3	3	0	95	101	−0.12	1	5.90	9.30 ± 2.30	6.00 ± 2.00
Myanmar	n/a	n/a	n/a	n/a	73	1.00	n/a	19.20	14.50 ± 10.70	3.60 ± 2.70
SW Yunnan, China	0	0	5	43	48	1.00	<0.0001	10.40	2.40 ± 0.90	2.40 ± 0.90
Tongbiguan, Yunnan, China	0	0	10	533	543	0.95	<0.0001	2.00	13.70 ± 5.20	12.40 ± 5.30
Nanling Mountains, China	0	0	98	243	341	1.00	<0.0001	28.70	11.60 ± 4.10	3.70 ± 4.50
Zhejiang, China	3	0	5	140	148	0.78	<0.0001	5.40	6.20 ± 3.90	10.20 ± 6.50

### 3.3. Association of the species in flocks of Nepal and their foraging ecology

When the species were observed together, the average distance between the species was 4.16 ± 2.99 m. There were no observations of aggressive behaviour between the species except twice (two different flocks on different transects) when a GNLT displaced the feeding site of a LNLT. We saw a total of 44 instances on six transects in which GNLTs were followed by LNLTs and only four instances on four transects in which LNLTs were followed by GNLTs.

**Figure 3.**
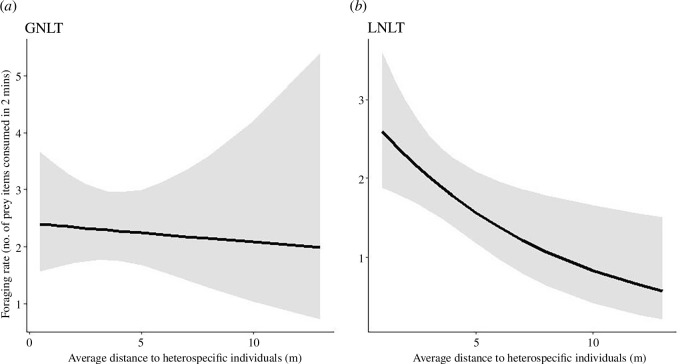
(*a*, *b*) The foraging rate of NLTs related to the distance to a heterospecific in MSFs. The foraging rate was defined as the number of food items consumed in 2 min. Graphs show partial residual graphs, evaluating the influence of heterospecific distance to foraging success when conspecific distance was kept constant. The grey-shaded area represents estimates within the 95% confidence interval.

The species did not differ in the spacing between conspecifics when they were in a flock by themselves compared to a flock in which the other species was present (for GNLT: 0.98 ± 0.29 m apart in monospecific flocks, 1.92 ± 2.13 m in MSFs with LNLTs, *ß* = −1.02 ± 0.57, *p* = 0.10); for LNLT: 1.21 ± 1.09 m in monospecific flocks, 0.94 ± 0.67 m in MSFs with GNLTs, *ß* = 0.37 ± 0.31, *p* = 0.23). Nor did species’ foraging success rates vary depending on whether they were in single or mixed flocks (for GNLT: 3.00 ± 1.53 foraging success per 2 min in monospecific flocks, 2.33 ± 1.69 in MSFs with LNLTs, *ß* = 0.26 ± 0.18, *p* = 0.15); for LNLT: 2.69 ± 2.24 in monospecific flocks, 1.85 ± 1.27 in MSFs with GNLTs, *ß* = 0.21 ± 0.19, *p* = 0.28). When the species were together, the foraging success rate of LNLTs was reduced when their distance to GNLTs was increased (*ß* = −0.13 ± 0.05, *p* = 0.011; figure 3). The foraging rate of GNLTs was not significantly affected by distance to LNLTs, *ß* = −0.02 ± 0.05, *p* = 0.78). Neither species’ foraging rate was affected by its distance to conspecifics.

## 4. Discussion

### 4.1. Evaluation of plumage and morphological similarity in relation to co-occurrence

We found weak evidence from museum specimens that the smaller LNLT is imitating the larger GNLT, in accordance with the predictions of ISDM theory. The brightness of LNLT’s necklace was more similar to GNLT when the two species were sympatric, and the colour of allopatric LNLTs was particularly variable, as might be expected for an imitating species in the absence of the model. LNLTs became darker in colder areas, which is opposite to the pattern expected for Gloger’s rule (although there may be a stronger relationship to humidity than to latitude [36]), favouring an explanation for the change different from environmental conditions. At the same time, there remains considerable variation in both species’ plumage colour in sympatry, and the clinal analysis shows that GNLT may also change across latitude, so our conclusions should be considered preliminary. The idea that LNLTs may be changing their appearance more than GNLTs supports the ISDM hypothesis—or mimicry of dominant competitors to fool third parties—as opposed to similarities between species facilitating communication [22] or an oddity effect that confuses predators [23], in which the two species might be expected to both change to resemble each other (see table 1).

Surprisingly, sympatric LNLTs were actually less similar to GNLTs in the proportional size of the necklace to the overall body than allopatric LNLTs. One explanation for this result could be that it is related to the dominance relationship between the species. The necklace has some similarities to a ‘badge of dominance’ that many birds display [46,47]. Therefore, it is possible, though admittedly speculative, that LNLTs with smaller necklaces would have a fitness advantage in places where they associate with GNLTs, reducing aggression by appearing more submissive.

LNLTs also show some changes in size across latitudes. Their tarsi are smaller in sympatry with GNLTs. Tarsus length is often associated with overall size of birds [48]; in decreasing in colder regions, the pattern for LNLT tarsi is opposite that of Bergmann’s rule [34], again favouring an explanation other than environmental change. It is possible that this could be an indication of character displacement, and hence competition, between the two species which is also consistent with the idea that ISDM could be operating in this system. However, character displacement in birds is most often found in beak size and not tarsus size [30,49]. Smaller tarsi can be an indication of less foraging on the ground [50], so this could be an indication that LNLT are competitively forced to shift their vertical foraging niche when in sympatry with GNLT. Yet, again, the data are non-conclusive. For example, there is some evidence against the idea of LNLTs becoming generally smaller, and GNLTs becoming generally larger in sympatry (the clinal analysis showed LNLT body size actually increasing in sympatry, and the *t*-tests showed sympatric GNLTs had smaller tails). Therefore, this result could also be owing to random variation, and we should emphasize that our allopatric populations had small sample sizes.

### 4.2. Co-occurrence of the species in mixed-species flocks

The co-occurrence data demonstrate that whatever interaction occurs between these two species, it occurs when they are grouped together in MSFs. Only one of six surveys analysed (that in Arunachal Pradesh, India) lacked a significant tendency for the two species to be together; and in that survey, the total number of MSFs in which the two species were seen was small (six MSFs). In two surveys, the two species were never seen apart, so it is fair to say that whatever the interaction between the species is, it is quite strong.

There are few other interesting patterns to be seen in the MSF data. These are not very abundant species anywhere: even in the system where they were most common, they were in fewer than 36% of the MSFs in the area. NLTs in Myanmar are members of an MSF system that includes other large species such as drongos, magpies and treepies [38]. These species were also occasionally seen with NLTs in Nepal, in the Nanling mountains and in Zhejiang Province, China. So, it is possible that NLTs are more common in MSFs that specifically include large-bodied species. Finally, the data show that there is extensive variation in the numbers of individuals of the two species. In some systems, the numbers of individuals of LNLT are much greater than the numbers of GNLT, but in other systems it is the opposite.

### 4.3. Association of the species in flocks of Nepal and their foraging ecology

Some aspects of the species’ behaviour in Nepal suggest that the species do not benefit equally from the interactions. LNLTs clearly followed GNLTs and had a significant relationship between foraging success and distance to GNLTs. The mechanism behind how distance between species might affect feeding rates was not clear in the field. There was not an obvious connection to beating, the phenomenon in which the movement of birds disturbs insects into the air, where they can be caught [51–53]: the birds were not directly above or below each other. Nor did we see evidence of the birds simultanously eating clustered prey items in an MSF.

More likely, foraging benefits may come from species closer together having less of a need to be vigilant, and therefore being able to concentrate more time on foraging [54]. This could be the result of a number of mechanisms: (i) an oddity effect, as mentioned in the introduction; (ii) benefits from eavesdropping on heterospecific alarms [55], which are very frequently made by GNLT (KR Gosai 2021, personal observation); and (iii) it could be that GNLTs are aggressive or noxious to predators, and LNLTs benefit from being proximate to them and similar in appearance, thus reducing their own risk of attack [20]. In all these scenarios, the mimicry could allow LNLTs to be closer to the dominant GNLTs than would otherwise be allowed.

One major criticism of ISDM is that birds are often able to individually recognize conspecifics through visual differences [56] and therefore it would seem unlikely that they would be fooled by heterospecifics that appear only somewhat similar to conspecifics [19]. Against this argument in the case of the NLTs, we would like to emphasize that the birds are actively moving in thick vegetation with low amounts of light. In some situations, birds may only be in direct visual contact with the closest neighbour sporadically, as they move between and behind leaves, focusing their visual attention on their prey. We cannot rule out the possibility that the mimicry of LNLTs is directed to third parties. However, we find it less likely than ISDM for two reasons. First, these two species are their closest associates (indeed, in some places like the Nanling Mountains of southern China, they are usually in two-species MSFs), so it is unlikely that this mimicry is duping some other competing species. Second, if the deceived party is a predator, there is no connection between the close association of the two species and their mimicry.

### 4.4. Limitations and future directions

Despite trying to collect many aspects of data on these species, we feel as if we have not yet definitely cracked the puzzle of their association, and there are many aspects that we hope future studies can emphasize. First, we do not demonstrate that these two species truly look more like one another than expected based on phylogeny, ecology, or other factors, and this could be a key next step for research. Specifically, about the plumage resemblances, we did not test aspects of plumage mimicry other than the necklace, yet it seems that multiple parts of the plumage are similar between the two species (see figure 1). As mentioned above, sample sizes for allopatric populations were low; we classified some small potential patches of allopatry in Yunnan (as indicated in ) as sympatry, because we were unsure of the precision of the data that produced the BoW distribution maps. Finally, our behavioural work was only done in one system, in which there were more LNLTs than GNLTs. However, the different systems vary a lot from each other and therefore observations on foraging and spacing need to be studied in other systems. Studies of the flocking behaviour of the species in allopatry (central eastern China for GNLTs and southern Vietnam for LNLTs) would also be very interesting.

Further work on the system could also extend the investigation into different aspects and functions of mimicry. Vocal characteristics should also be investigated, as the two species can sound quite similar in some contexts (L Zhou, personal observation). We did not test whether the feathers or skin of either species was distasteful or poisonous, and given the findings in Papua New Guinea [13], toxicity or mere distastefulness cannot be dismissed [18] and should be further explored.

## 5. Conclusion

We studied an interesting case of MSF-associated mimicry, in which two species associated together in MSFs resemble each other, although they are not close relatives (i.e. not in the same genus). Several findings suggest that this phenomenon is related to dominance: the smaller species (LNLT) appears to change to match the plumage colour of the dominant (GNLT) in sympatry, is more variable in allopatry, and has proportionally smaller necklaces in sympatry, which could be a badge of dominance; in Nepal, LNLTs follow GNLTs and appear to benefit more from the relationship, foraging more when closer to GNLTs. Given that the species are often in quite close proximity in MSFs in dense vegetation and low light conditions, it seems likely that in some situations the ‘dupe’ (the audience for mimicry) [57] might be GNLTs, which would treat LNLTs with less aggression than otherwise, similar to the relationship between friarbirds and orioles [15,58] and consistent with the idea of ISDM [16]. However, some parts of our results did not match this hypothesis (e.g. foraging of NLTs was not higher when they were together in MSFs than when they were separated), and there are many more aspects of mimicry to investigate in this system in future.

## Data Availability

The datasets supporting this article have been uploaded as part of the electronic supplementary material [32].
